# The Impact of a Filariasis Control Program on Lihir Island, Papua New Guinea

**DOI:** 10.1371/journal.pntd.0001286

**Published:** 2011-08-23

**Authors:** Oriol Mitjà, Raymond Paru, Russell Hays, Lysaght Griffin, Nedley Laban, Mellie Samson, Quique Bassat

**Affiliations:** 1 Department of Medicine, Lihir Medical Centre, International SOS, Lihir Island, New Ireland Province, Papua New Guinea; 2 Department of Public Health, Lihir Medical Centre, International SOS, Lihir Island, New Ireland Province, Papua New Guinea; 3 Department of Microbiology, Lihir Medical Centre, International SOS, Lihir Island, New Ireland Province, Papua New Guinea; 4 Barcelona Centre for International Health Research, Hospital Clínic, University of Barcelona, Barcelona, Spain; Centers for Disease Control and Prevention, United States of America

## Abstract

**Background:**

Annual mass drug administration (MDA) over five years is the WHO's recommended strategy to eliminate lymphatic filariasis (LF). Some experts, however, consider that longer periods of treatment might be necessary in certain high prevalence and transmission environments based upon past unsuccessful field experience and modelling.

**Methodology/Principal Findings:**

To evaluate predictors of success in a LF control program we conducted an ecological study during a pre-existing MDA program. We studied 27 villages in Lihir Island, Papua New Guinea, from two areas with different infection rates before MDA. We undertook surveys to collect information on variables potentially having an influence on the outcome of the program, including epidemiological (baseline prevalence of infection, immigration rate), entomological (vector density) and operational (treatment coverage, vector control strategies) variables. The success in a village was defined using variables related to the infection (circulating filarial antigenemia prevalence <1%) and transmission (antigenemia prevalence <1 in 1000 children born since start of MDA). 8709 people were involved in the MDA program and average coverage rates were around 70%. The overall prevalence of filariasis fell from an initial 17.91% to 3.76% at round 5 (p<0.001). Viewed on a village by village basis, 12/27 (44%) villages achieved success. In multivariate analysis, low baseline prevalence was the only factor predicting both success in reducing infection rates (OR 19,26; CI 95% 1,12 to 331,82) and success in preventing new infections (OR 27,44; CI 95% 1,05 to 719,6). Low vector density and the use of an optimal vector control strategy were also associated with success in reducing infection rates, but this did not reach statistical significance.

**Conclusions/Significance:**

Our results provide the data that supports the recommendation that high endemic areas may require longer duration MDA programs, or alternative control strategies.

## Author Summary

Large-scale intervention programmes to control filariasis are currently underway worldwide. However, a major unresolved question remains: what is the appropriate duration for these programmes? Recent theoretical work and clinical field experience has highlighted how the ecological diversity between different endemic regions hinders decision making processes of when to stop ongoing MDA programs. The goal of our study was to identify the factors determining success for a five year LF elimination program. We undertook different types of surveys together with a pre-existing MDA program in villages from two regions that had different infection prevalence rates. Our study shows that the five yearly cycles of MDA could neither eliminate the disease nor stop transmission in the high prevalence villages, such that low baseline lymphatic filariasis prevalence has a positive influence on the outcome of a program. Thus, the study provides data supporting the recommendation that in certain high prevalence and transmission environments more sustained efforts may be necessary.

## Introduction

Lymphatic filariasis (LF), caused by the mosquito-borne nematode Wuchereria Bancrofti, is a major public-health problem in many tropical and subtropical regions. Papua New Guinea represents the biggest remaining challenge for elimination of the disease. The Global Program to Eliminate Lymphatic Filariasis (GPELF) was launched in 1997. In the Pacific, the World Health Organization (WHO) has implemented from 1999, the Pacific Program to Eliminate Lymphatic Filariasis (PacELF) bringing together 22 countries and territories, in a common effort to eliminate the disease [1], [2]. The PacELF strategy is based on five rounds of mass drug administration (MDA), monitored by a prevalence survey to assess the impact at completion of the last round [3], [4]. Therefore, the assessment is designed to conclude whether to stop or to continue MDA after round 5. The rationale of this approach is to suppress microfilaremia (mf) in infected populations and bring the infection level down below a threshold that will prevent resurgence of infection and ultimately lead to interruption of transmission [5].

The exact infection level to achieve LF elimination in different endemic regions remains unknown, such that it is difficult to predict or decide when to stop ongoing MDA programs. Previous reports have suggested that residual filarial infections disappear when prevalence rates fall to less than 1% but it may vary depending on specific ecological conditions [6], [7]. Moreover, some programs which have achieved this threshold have reported evidence of ongoing transmission, as measured by antibody or antigen prevalence in children aged 2–4 years and mosquito infection rates [8], [9]. The current recommendation of the PacELF is that programs should reach an antigenemia level below 1% and that less than 1 in 1000 children born since start of MDA should become newly infected [1], [2]. End-points for the GPELF have recently been changed to a level below 2% in areas where the main vector is an anopheline [4].The optimal duration of MDA programs has also not been established. Mathematical models suggest that 4 to 6 years of treatment should be sufficient [10]. However, several programs have reported evidence of failure to control the infection, as indicated by mf and circulating filarial antigenemia prevalence rates after completing five annual rounds of MDA [11]–[14].

Numerous attempts have been made to establish which variables may influence the outcome of a program. Some variables, such as the coverage of the target population [9], [15], [16], the drug regimen employed [5], [17]–[20], and the integration of vector control measures [21]–[23], are controllable. Other biological and epidemiological variables, such as the initial prevalence of mf in an area and the vectorial abundance of the mosquito, are less amenable to modification. All the above mentioned factors need to be taken into consideration when developing an elimination strategy [24].

The aim of this study was to estimate success rates of the program to eliminate lymphatic filariasis (PELF) in villages from different areas and to identify determinants of success affecting a PELF's outcome.

## Methods

Data for this ecological study were collected at a community-level during the delivery of an MDA program in villages of Lihir Island, Papua New Guinea. The program was closely monitored epidemiologically, entomologically, and through laboratory studies as outlined below. Together with drug administration we undertook different types of surveys including village surveys to collect information about variables potentially having an influence on the outcome of the program; circulating filarial antigenemia (CFA) prevalence surveys to assess the infection status and new infections since start of MDA; and mosquito surveys to determine mosquito abundance. CFA prevalence of the entire population was reassessed once after round 5.

The surveys were administered to the entire population of 27 villages in two regions of Lihir Island that had different infection prevalence rates before MDA was initiated. The study villages are only separated by between 1 and 3 kilometres from each other, however they constitute independent transmission zones as the vector species, *Anopheles farauti*, is generally considered incapable of flying more than 700 metres [25]. Many residents had been previously treated with diethylcarbamazine citrate (DEC) alone in 1995 during a campaign started by the New Ireland provincial government which continued for a short time [25]. However, ten out of twelve villages located in the swampy regions of the west coast recorded prevalence levels of filariasis as high as 20–60% in 2003. The other study villages on the dry savannah grassland of the north-east had rates 3–7 times lower than those in the west. In addition, the eastern area of the island in the vicinity of Londolovit has been host to a mining operation by LGL Australia since 1996. This has seen the influx of approximately 2000 workers from other areas of PNG and internationally, and a considerable unofficial migrant population from surrounding regions. There has been a degree of local development in the mine affected area.

Field teams consisting of a physician, a technician, and a local health worker visited the villages at spot check sites, annually. Prior to each visit, efforts were made to educate the public to the program's aims through health educators who disseminated information about LF at community meetings and through public notices, in cooperation with church and village leaders. MDA comprised the WHO-recommended regimen of a single oral dose of DEC (6 mg/kg body weight) and albendazole (400 mg regardless of weight) under direct observation. Simultaneously the teams collected the blood samples and recorded on a register epidemiological data and coverage information. Some people agreed to receive MDA but refused to provide blood samples. The observed coverage rates were based on the number of subjects seen to ingest the tablets and calculated on the basis of the total eligible population. However, a coverage survey was not carried out to verify the coverage achieved. The global programme uses coverage data reported by surveys, while PacELF uses data from registers [1]. The source of data chosen to calculate the total population was the local census carried out among all island villagers annually. We classified as high drug coverage rate all villages where the percentage of treated population was estimated at more than 70%. Migration from other areas was considered to be low when it involved less than 5% of the village population.

Circulating filarial antigenemia (CFA) was assessed with a rapid-format antigen card test (Filariasis Now, Binax Inc., Portland, Maine, USA) together with the first and after fifth round of MDA. While microfilaremia is the gold standard for monitoring filarial infection, PacELF guidelines are based on the use of the antigen test in a community-wide survey [1], as this is a simple card test with a high reported sensitivity (98.5%) and specificity (100%) [26], [27], and provides the additional advantage of allowing daytime blood sampling. We defined the prevalence rate of CFA as the number of people with a positive antigen test divided by the number of people tested. A low endemicity was defined as a CFA positivity rate in the population of less than 10%.

Mosquitoes were collected to assess the vector biting activity before the first round of MDA (January 2003). Indoor human landing catches of the vector mosquito, *Anopheles farauti* [25], were conducted from sunset to sunrise (18:30–06:30 h). With the help of a simple aspirator mosquitoes were caught when they landed on a human volunteer for taking a blood meal. A monthly biting rate (MBR) was computed by multiplying the number of mosquitoes contacting a man per 24 hours with the number of days in the month. Vector density was considered to be low when the MBR was under 100 bites/person/month.

A supplementary vector control strategy involving anti-mosquito measures was put in place. Source reduction of potential breeding sites in the vicinity of villages, and community-wide distribution of long-lasting insecticide-treated netting materials (LLITNs) were promoted in the entire island. However, indoor spraying of residual pyrethroids was used only in certain villages located in the vicinity of the Lihir Gold mine. We defined an optimal vector control strategy as one where all of these anti-mosquito tools were employed. Our study did not assess compliance in the use of bed nets, nor assess the effectiveness of local mosquito control measures.

The success of the program in a village was defined using variables related to the infection (reaching antigenemia level below 1%) and transmission (less than 1 in 1000 children born in the community since start of MDA having infection measured by antigenemia).

### Statistical analysis

Data entry was undertaken with EpiInfo software (version 6), with field limits and double data entry. We analysed demographic data (age and sex), migration from other areas, the initial endemicity of infection (prevalence rate), the vector abundance, treatment coverage (number of tablets distributed), the use of adjuvant vector control strategies and the outcome of the elimination program. Univariate analyses of data from the villages were performed using the *χ* ^2^ test for categorical data, and the *t*-test or Mann–Whitney *U*-test for continuous data. Predictors for success of PELF in controlling the infection prevalence were analysed by multivariate logistic regression, which included the following variables: low baseline prevalence, low migration, low vector density, high treatment coverage, and optimal vector control strategy. Predictors of success in stopping the transmission were also analysed by logistic regression analysis, which included the variables: average age <20 years, low endemicity of infection, low vector density and high treatment coverage. Odds ratios and 95% confidence intervals are presented. All multivariate logistic analyses were done using Firth's method to overcome problems of separation in small samples [28]. Data were analysed using SAS 9.1.3 (SAS Institute Inc., Cary, NC, USA) and SPSS 14.0 (SPSS Inc., Chicago, IL, USA).

Reporting of the study has been verified in accordance with the STROBE checklist (provided as Checklist S1).

### Ethical clearance

Ethics approval for this study, including the oral consent process, was obtained from the Papua New Guinea Ministry of Health Medical Research Advisory Committee. The consent sought was verbal because of the high illiteracy rate in rural population, and it was documented on case report forms. Study personnel informed prospective study participants about the study by reading them a consent document in the local language. All subjects provided informed consent at every stage of the study including for collection of samples, interviews, and individuals involved in calculating monthly bite rates. Participation by children required consent from at least one parent and the child's assent.

## Results

The annual census in the year 2003 estimated that 8709 people lived in the 27 villages which were part of the study and that were visited and treated annually over a period of 5 years. At the baseline survey, a total of 6037 individuals were registered corresponding to 70.0% of the entire population (as determined by the 2003 census). 50% of the villagers were male, and the mean age was 20.6 years. Reported coverage in 2004–2007 for MDAs 2–5 was 69.8%, 73.0%, 74.1% and 71.5% respectively. Drug coverage remained stable over time and it was similar in all the territories, with an average of 72,9% in eastern coast villages and 67,4% in western coast villages (p = 0.35). The reason why 26% to 30% of the target population were not treated is that they were not available at the time of the medical team visit (i.e. working, visiting relatives) or that they were ineligible persons (3.0% to 6.0% of the de facto population), including pregnant women and children younger than 2 years old or of weight less than 10 kg.

**Figure 1:**
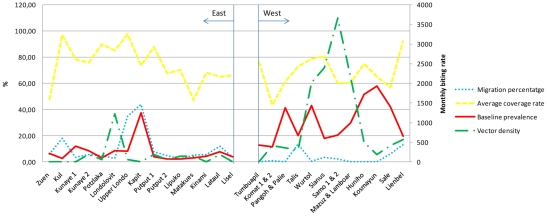
Baseline epidemiological, infection status and entomological data and program coverage details of 27 villages, Lihir Island.

The demographic and epidemiological data and program operational details of the 27 villages are shown in Figure 1. Almost half (44,4%) of the villages had migration rate over 5% of the total population, 12 (80.0%) of them were villages from the eastern coast. The migrants mainly came from low endemic areas for filariasis such as the PNG mainland, and from the small islands surrounding Lihir. The details of treatment coverage were recorded during all the five rounds of treatment and the average calculated. Individually, 55.6% of the villages had a high drug coverage, including 60,0% (9/15) in the eastern coast group and 50,0% (6/12) in the western coast group.

Mosquito transmission indices varied significantly in different villages. The highest indices of transmission were observed in villages located in the swampier regions of the west coast with a MBR median (interquartile range) of 460.8 (1812.6) bites/person/month, compared to 57.6 (180.0) in the east coast. These data indicate that there is regional micro-variation in the intensity and temporal pattern of filariasis transmission. It is noteworthy that only nine villages (33.0%) used indoor residual spraying and therefore achieved an optimal vector control strategy.

Table 1 shows data for filarial infection rates before and after the PELF. Overall, all variables showed significant decreases from pre-MDA to round 5 (p<0.001). The analysis of data from all villages shows a significant decrease in circulating filarial antigenemia (CFA) over this period from a mean prevalence of 17.9% to 3.8% (p<0.001). Pre-MDA CFA prevalence rates were much higher in the Western than in the Eastern territories. The mean prevalence of infection was 7.7% and 0.8% in eastern villages, and 30.7% and 7,5% in western villages in the pre-MDA and at completion of round 5, respectively (table 1). Pre-MDA antigenemia prevalence rates in children under 5 years were not significantly different in the western village schools and in the eastern village schools (60.1 vs 31.0 in 1000; p = 0.10). Rates of circulating filarial antigenemia in under 5 y.o. children fell more rapidly in the less heavily infected eastern villages than in the western villages (30.0 vs 0.95 in 1000; p<0.01). Twenty-one children with a positive result out of 700 tested were identified in the Western villages after round 5.

**Table 1:** Effect of MDA on circulating filarial antigenemia prevalence rates on Lihir Island.

	Infection prevalence		Ongoing transmission (new infections detected)	
	Number of people tested	Antigenemia prevalence rate^a^ (%[SD])	Number of children tested	Positive Antigenemia in 1000 children^b^ (%[SD])
East coast study area				
Pre-MDA	3009	7.67 (8.77)	537	30.98 (49.19)
MDA round 5	3799	0.76 (0.92)	1006	0.96 (3.68)
West coast study area				
Pre-MDA	1969	30.71 (15.87)	462	60.14 (37.36)
MDA round 5	2464	7.51 (3.77)	700	30.00 (29.96)
Total				
Pre-MDA	4978	17.91 (16.86)	999	43.94 (45.95)
MDA round 5	6263	3.76 (4.26)	1706	13.86 (24.57)

a Antigenemia prevalence rate in a large size sample from the whole treated population. Some people agreed to receive MDA but refused to provide blood samples.

b Antigenemia prevalence rate in children under 5 years old which represents new infections since start of MDA.

**Figure 2:**
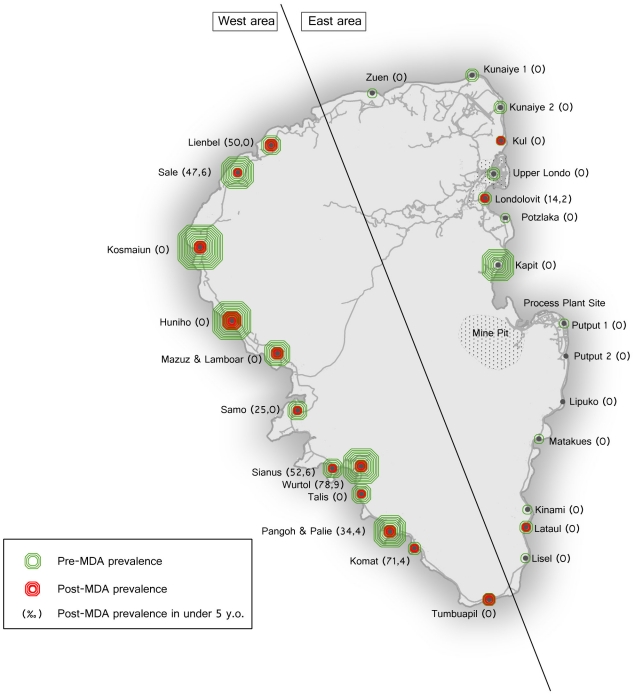
Percentage of Infected people, in Lihir Island Villages, in the pre-MDA and post-MDA surveys. NOTE. The diameter of the circles is proportional to the estimated prevalence of Lymphatic filariasis, implying that every line corresponds to an increase in prevalence of 5%.

As shown in figure 2, the CFA prevalence in individual villages ranged from 1.1% to 58% and 0 to 17%, in the pre-MDA and post-MDA surveys respectively. PELF had a successful outcome on infection prevalence control in 12 of the 27 villages (44.4%), whereas it failed in the remaining 15 (55.5%). Transmission, assessed on the incidence of new infections, was successfully controlled in 19 (70,3%) villages. On univariate analysis (table 2), numerous factors were found to be significantly associated with PELF success on the control of infection status including low baseline prevalence, low vector abundance, and implementation of an optimal vectorial control. A low percentage of migration, unexpectedly, was found to be a risk factor. On multivariate analysis (table 2), the only independent factor predicting PELF infection control success was low endemicity of infection (OR 19.26; CI 95% 1.12–331.82). Table 3 shows the univariate and multivariate predictors associated with transmission control success. Low endemicity of infection (OR 27.44; CI 95% 1.05–719.6) was again the only factor independently associated with transmission control success.

**Table 2:** Association between characteristics of villages and program related factors and PELF success to control infection prevalence.

Factor	Villages where PELF succeeded(N = 12)	Villages where PELF failed(N = 15)	Univariate Analysis		Multivariate Analysis	
			OR (95% CI)	P value*	OR (95% CI)	P value*
Demographic data						
average age <20 yr old	3 (25.0)	6 (40.0)	0.5 (0.09–2.64)	0.41		
sex, male-female rate >0.50	7 (58.3)	7 (46.7)	1.60 (0,34–7.40)	0.54		
Epidemiologic data						
Low endemicity of infection	10 (83.3)	3 (20.0)	20.0 (2.77–144.31)	0.003	19.26 (1.12–331.82)	0.04
Low migration from other endemic areas	2 (16.7)	10 (66.7)	0.1 (0.02–0.64)	0.015	0.57 (0.03–11.46)	0.72
Low vector density	8 (66.7)	2 (13.3)	13.0 (1.92–87.99)	0.009	11.58 (0.68–197.0)	0.09
Operational data						
High treatment coverage	7 (58.3)	8 (53.3)	1.22 (0,27–5.67)	0.79	0.23 (0.01–11.28)	0.46
Optimal vectorial control	7 (58.3)	1 (6.7)	19.60 (1.91–201.62)	0.01	18.00 (0.36–894.6)	0.14

OR, odds ratio. PELF, Program for Elimination of Lymphatic Filariasis.

*A P value ,0.05 was considered to be statistically significant.

**Table 3:** Association between characteristics of villages and program related factors and PELF success to stop transmission.

Factor	Villages where PELF succeeded(N = 19)	Villages where PELF failed(N = 8)	Univariate Analysis		Multivariate Analysis	
			OR (95% CI)	P value*	OR (95% CI)	P value*
Demographic data						
average age <20 yr old	6 (31.6)	3 (37.5)	0.76 (0.13–4.32)	0.76	3.19 (0.21–46.86)	0.40
sex, male-female rate >0.50	9 (47.4)	5 (62.5)	0.54 (0.1–2.93)	0.47		
Epidemiologic data						
Low endemicity of infection	12 (63.2)	8 (12.5)	12.00 (1.21–118.89)	0.03	27.44 (1.05–719.6)	0.04
Low migration from other endemic areas	7 (36.8)	5 (62.5)	0.35 (0.06–1.93)	0.23		
Low vector density	9 (47.4)	1 (12.5)	6.30 (0.64–61.63)	0.11	21.85 (0.61–786.3)	0.09
Operational data						
High treatment coverage	9 (47.4)	6 (75.0)	0.30 (0.05–1.88)	0.20	0.08 (0.01–1.41)	0.08
Optimal vectorial control	7 (36.8)	1 (12.5)	4.08 (0.41–40.45)	0.23		

OR, odds ratio. PELF, Program for Elimination of Lymphatic Filariasis.

*A P value ,0.05 was considered to be statistically significant.

## Discussion

In the present study the overall prevalence of circulating filarial antigenemia was reduced by 79.0%. Despite undeniable success, the program did not achieve its very ambitious goals based on the PacELF recommendations, with post treatment prevalence remaining at 3.8% and 14 new infections per 1000 children born since start of MDA. This overall failure was the result of the intervention specifically failing in some villages. Understanding what factors lead to success or failure when the intervention is applied to a specific setting may help improve the MDA program. Our findings reveal that in Lihir, the baseline infection status was an important factor influencing on the outcome of the PELF. Our discussion will focus on possible explanations for this observation and the influence of other factors on the outcome.

The first objective of our study was to test the hypothesis that bancroftian filariasis can be eliminated from communities by yearly cycles of MDA with diethylcarbamazine and albendazole. Over all, success in controlling the infection and in stopping transmission was confirmed in 45% and 70% of the villages, respectively, most of them located in the eastern coast. The eastern coast villages had low baseline levels of filariasis endemicity, whereas the western coast villages had very high baseline rates that were more typical of filariasis endemic islands in the Western Pacific Region [2]. Our data suggest that five rounds of MDA will not have eliminated filariasis in the western study area. However the observed post-treatment antigen prevalence rates are unlikely to sustain transmission as the vector-parasite relationship in Lihir is extremely fragile involving the *An. Farauti* which is one of the least efficient vectors in the world. The cut-off point for interruption of transmission in the PacELF region was based on the fact that transmission in most of the Pacific island countries is carried out by the highly efficient Culex and Aedes mosquitoes. The second major objective of our study was to evaluate the factors having a positive influence on the markers of success. In our study the most prominent determinant of success was low baseline prevalence of infection. Low vector density appeared to have an association but did not reach statistical significance. The sample size, as it is based on the number of villages, is unfortunately low and may have caused the study to be underpowered when trying to determine the significance of this trend. Other specific factors have previously been described as having a positive influence in the outcome of a PELF. These include high coverage of the targeted population [9], [15], [16], low levels of migration from other areas and integration of the different available control strategies into the program [21], [22]. In the current study however, these variables did not show an association.

Prior to the current study there has been little reliable clinical evidence comparing low prevalence communities with high prevalence populations. Our study followed a large cohort, contained detailed information about risk factors and outcomes and established comparisons among several independent areas of transmission. The current study also had the advantage of being conducted in an area of Papua New Guinea with a relatively high rate of infection and transmission, and a pronounced inter-area variation in prevalence. Moreover, an island population such as Lihir presents more ideal conditions for epidemiological studies and evaluation of control programs than large land areas. This study has the limitations of an observational ecological study, and obviously causality cannot be inferred from it. The observed risk factors need to be considered with caution. Also, data for some factors which may influence success rates (e.g. number of persons per household, use of bednets) were not collected. However, the analyses performed controlling for the widely recognized prime predictors, the strong and similar results obtained in the multivariate analyses for two different markers of success, and the use of specific techniques to obtain unbiased risk estimates with small samples allow us to have confidence in the results obtained.

The percentage of the population covered is an important factor in determining the success of a PELF that has been previously analysed [9], [15], [16]. We developed a timely and coordinated drug delivery strategy that included elements of community information and education in an attempt to achieve widespread acceptance of drug treatment and the DOT (Directly Observed Therapy) distribution method. We made an effort to reach those groups of individuals who are recognized to be at risk of systematic non-compliance during MDAs including children, the upper socioeconomic classes, young men and the elderly. We achieved an overall 70% reported population treatment coverage which was probably underestimated, since it was calculated using the number of people in the local census. In the multivariate analysis the coverage was not associated with any of the markers of success. This is likely due to the similar levels of coverage achieved in all the villages in the current program.

The lack of influence of population migration in this study may be explained by the demographics of the migrant population. The presence of the gold mining operation at Lihir has attracted a large number of relatively skilled and affluent workers, often from areas such as Port Moresby and other regional centres where LF endemicity is low. Thus migration did not contribute to the reservoir of filariasis in Lihir.

The long term impact of MDA is determined by the drugs' effects on microfilaria, and particularly on adult worms [29]. A single dose of DEC and Albendazole rapidly reduces the number of circulating microfilaria, but also has a temporary effect in reducing the production of microfilaria by adult worms, probably due to sterilization. After some months renewed production occurs but at a reduced intensity [20], [29]. In the absence of macrofilaricidal activity, current programs rely on the interruption of transmission through sustained suppression of microfilaremia over the 5 years of a program. Experimental studies have documented the median fecund lifespan of *W. Bancrofti* worms to be more than the 5 years typical of an MDA [30]. In addition, single doses of DEC and Albendazole have been shown to have a limited capacity to kill the adult worm. A Brazilian study found a significant proportion of adult worms were insensitive to DEC at doses of 6 mg/kg, with ultrasound studies showing only a 56% mortality for adult worms after 5 years [29]. This has led some physicians to suggest that MDA programs should be of at least the same duration as the lifespan of adult worms. Moreover, it has been demonstrated that individuals in areas of high endemicity will have a higher average adult worm burden, and therefore a higher chance of a fecund worm pair surviving after MDA is complete [14]. A theoretical analysis with field data from 9 villages, in distinct endemic areas, identified the degree of infection aggregation as one of the main factors related to failure of a PELF. Through a simulation procedure, the author estimated that following the current approach, only 50% of programs would achieve parasite elimination [6]. Dunyo et al. in their study showed a higher level of microfilarial resurgence than in other programs, and suggested that this may have been due to a high pre-treatment worm burden [31].

The fact that successful elimination of disease in high prevalence areas may require longer duration of MDA programmes has already been recognized by experts [1]–[4], [24], [32]–[34]. This paper provides the data that support such recommendations. It is clear that local data needs to be taken into account when designing MDA programs. Alternative strategies may be needed, including modified drug regimens (e.g., biannual MDA), vector control measures, or perhaps antibiotic treatment.

## Supporting Information

Checklist S1 STROBE checklist.
(DOC)
